# Editorial: Arboviruses: co-circulation, co-transmission, and co-infection

**DOI:** 10.3389/fmicb.2023.1321166

**Published:** 2023-11-07

**Authors:** Rúbens Prince dos Santos Alves, Jaime Henrique Amorim

**Affiliations:** ^1^Center for Infectious Disease and Vaccine Research, La Jolla Institute for Immunology, La Jolla, CA, United States; ^2^Western Bahia Virology Institute, Center of Biological Sciences and Health, Federal University of Western Bahia, Barreiras, Brazil

**Keywords:** co-transmission, arboviruses, co-circulation, co-infection, vectors

Arthropod-borne viruses, or arboviruses, have risen rapidly and consistently. Urbanization, increased travel, and climate change have accelerated their global spread. Consequently, they have become a major concern for global public health, leading to a surge in disease outbreaks. One of the most significant examples of arbovirus co-circulation involves the four serotypes of dengue viruses (DENV1-4), which account for over 400 million infections annually, threatening nearly 4 billion individuals globally. To add more complexity to the situation, the emergence of chikungunya and zika viruses in dengue-prevalent areas has raised significant concerns. These six viruses are closely interrelated in urban environments, sharing humans as common hosts and the arthropod vector (primarily *A. aegypti*). Consequently, they are influenced by similar biological, ecological, and economic dynamics. Their geographical overlap, parallel seasonality, and infection rates have resulted in concurrent outbreaks in various global regions. Beyond DENV, zika, and chikungunya viruses (ZIKV and CHIKV, respectively), other lesser-studied arboviruses like yellow fever (YFV), West Nile (WNV), mayaro (MAYV), and Japanese encephalitis (JEV) viruses also co-circulate in urban and surrounding areas across the globe.

As the arbovirus burden increases, coinfections in urban areas will likely increase. Our knowledge about the nuances of these coinfections and their impact on human health is limited. The consequences of simultaneous infections, whether from multiple or single vectors transmitting various viruses, are unclear. Such complexities could significantly affect disease dynamics and transmission patterns. de França Cirilo et al. characterized the circulation of mosquito-borne viruses in Western Bahia, Brazil. Researchers enrolled 98 patients clinically diagnosed with dengue from March to June 2021. Serum samples were analyzed for CHIKV, MAYV, DENV, ZIKV, and YFV. The findings revealed that 45 patients were infected with CHIKV, 32 with DENV-1 serotype, and six with ZIKV; 15 tested negative for all arboviruses. Laboratory diagnosis is crucial for identifying arbovirus infections, as shown by the co-circulation of multiple arboviruses in Western Bahia in 2021. The widely distributed *Aedes* spp. mosquitoes are vectors for several arboviruses, and different virus species or strains of the same virus may exhibit differences in characteristics such as fitness, tropism, and vector competence. van Bree et al. have shown that this is especially important for Usutu virus (USUV), an African-origin mosquito-borne flavivirus transmitted by *Culex* spp. that has spread across Europe, leading to significant bird die-offs. Despite understanding its varied genetic lineages, the implications of co-infection and transmission efficiency of concurrent USUV strains in Europe are still uncertain. In comparing two USUV isolates, the Dutch isolate (USUV-NL) was consistently outperformed by the Italian isolate (USUV-IT) in various cell lines, especially in mosquito cells. While individual infections in *Culex pipiens* mosquitoes showed no competence difference between the isolates, co-infection revealed that USUV-IT hindered USUV-NL's infectivity and transmission.

Phleboviruses are transmitted to humans and animals by sandflies. They are relatively neglected and are not on the priority list of national and international public health agencies. It may change due to climate, environmental changes, and human activity. Birds participate in the natural cycle of various arboviruses in urban and rural settings. Toscana (TOSV) and Sicilian (SFSV) phleboviruses, transmitted by sandflies, can lead to human diseases, but the role of birds in their natural cycle is poorly understood. Ayhan et al. examining common quail sera from northern Spain, showed high neutralizing antibody rates for SFSV (45.45%) and TOSV (42.45%) were discovered, marking the first identification of these antibodies in wild birds. The significant seroprevalence in quails suggests they might play a role as amplifying hosts in the phleboviruses' natural cycle. Still, the often-overlooked public health issue of sandfly-borne infections is growing in Mediterranean countries and expanding northwards in Europe.

Cai et al. isolated Tahyna virus (TAHV) from *Aedes* sp. mosquitoes in Panjin City, Liaoning province. After oral infection, *Aedes albopictus* carried the virus in multiple tissues and had an incubation period of 2 days, indicating *Ae. albopictus* as an efficient vector and reservoir host for TAHV. Suckling mice were bitten by infected *Ae. albopictus* showed neurological symptoms. This discovery has epidemiological significance because, although low in incidence, DENV co-circulates in this region, and incidence may increase over time due to a temperature rise. Ticks are significant transmitters of arboviruses, and this region of China has a diverse tick population. By metagenomic analysis, Bai et al. examined more than 500 ticks from this area. They identified viruses related to human and animal diseases, including severe fever with thrombocytopenia syndrome virus (SFTSV) and Nairobi sheep disease virus (NSDV). The Dabieshan tick virus (DBTV) was particularly prevalent, showing a higher infection rate than in other Chinese provinces. Additionally, tick-borne viruses from the family Rhabdoviridae were identified in Liaoning Province for the first time. This research provides valuable insight into potential disease outbreaks in the region and the risks associated with tick bites. There is a geographic overlapping of these viruses and their hosts. In the sylvatic cycle of some of these viruses, small mammals serve as intermediate hosts for several arbo- and non-arboviruses; we have decided to include Du et al. study wherein in Jiangxi, China, hantaviruses and Wenzhou mammarenavirus concurrently circulate and co-infect both small mammals and humans. Although they are not arboviruses, recombination or reassortment with other negative- or positive-sense RNA arboviruses is possible.

In conclusion, the rapid surge in arthropod-borne viruses is a critical concern for global public health. Factors such as urbanization, climate change, and increased travel have expedited the spread of these viruses, with the dengue viruses being particularly prominent. Recent studies from various regions have highlighted the co-circulation of multiple arboviruses, with some, like the Usutu virus, presenting differential infection rates between strains. Equally concerning is the increasing recognition of sandfly-borne infections and the potential role of birds as amplifiers in the natural cycle of certain viruses. Additionally, findings from China highlight the diverse vectors transmitting various viruses, suggesting a complex interaction of hosts, viruses, and environmental factors. This accumulating data emphasizes the urgent need for comprehensive research to understand co-infections, transmission dynamics, and the broader implications of arbovirus co-circulation on human and animal health.

## Author contributions

RA: Conceptualization, Writing—original draft, Writing—review & editing. JA: Writing—review & editing.

